# Waiting outside the treatment room is not empty time: Family presence as supportive care in radiotherapy

**DOI:** 10.1017/S1478951526102685

**Published:** 2026-05-13

**Authors:** Hsiao-Chuan Liu, Yen-Ting Liu, Chao Yuan Tu

**Affiliations:** 1 Division of Radiation Oncology, Department of Oncology, National Taiwan University Hospital, College of Medicine, National Taiwan University, Taipei, Taiwan; 2 Division of Radiation Oncology, Department of Oncology, National Taiwan University Hospital Yunlin Branch, Yunlin, Taiwan; 3 Department of Biomedical Engineering, College of Medicine and College of Engineering, National Taiwan University, Taipei, Taiwan

In radiotherapy, family caregivers often walk patients to the treatment-room door and then wait outside the formal encounter. No procedure, image, or progress note is attached to that interval, so it is easy for staff to treat it as time outside care. From a supportive-care perspective, however, it is part of caregiving itself: repeated presence, uncertainty, and immediate availability when the patient comes back out. Applebaum (2022) argued that there is nothing informal about caregiving, and work published since then has continued to show that caregiver distress, anxiety, depression, and burden move with the patient’s illness course rather than sitting harmlessly outside it (Aubin et al. 2022; Valentino et al. 2026). When that experience remains unnamed, it usually remains unsupported as well.

What makes radiotherapy distinctive is not simply that family members wait. It is the routine practice that requires them to wait outside. Because of radiation safety, the patient enters the treatment room alone while the caregiver stays at the threshold. This is not a one-off waiting-room experience. It is repeated over fractions, over weeks, and sometimes across replanning, interruption, and changes in treatment intent. Each treatment day requires the caregiver to hand the patient over, tolerate not knowing what is happening inside the room, and resume practical and emotional care as soon as the patient comes back out. In many departments, the separation is so routine that staff hardly notice it. For families, however, routine does not automatically make it easy. The repetition matters. What may look minor in a single session can accumulate into a recognizable supportive care burden over the course of treatment.

In daily practice, what families often remember is not the machine itself but the moments around it. I still remember a husband who returned alone after his wife’s course had been discontinued and handed her worn treatment card back to the front desk. For the department, that was administrative closure; for him, it looked more like the end of a role he had carried every day. Such moments show that what happens at the threshold of the treatment room can carry meaning well beyond workflow. They are often where family members begin to make sense of illness, treatment, and loss (Teo et al. 2024).

Waiting outside the room should therefore be understood as clinical rather than merely logistical. The caregiver in that chair is often the first to notice that the patient is more tired after the fifth or tenth fraction, the first to hear what was not fully understood during the visit, and the person who carries the practical consequences of treatment home. That role often includes transport, meals, medication reminders, schedule changes, work adjustments, and the quiet work of deciding what should or should not worry the patient later that evening. Work published in this journal has linked caregiver experience to preparedness and communication (Bratches et al. 2024), the language used in difficult conversations (Miller et al. 2024), and whether caregiver concerns are recognized rather than assumed (Oliveira et al. 2024). Other recent studies have also emphasized caregivers’ contributions to dignity and meaning-making in serious illness (Teo et al. 2024; Sailian et al. 2025). In radiotherapy, many of those functions begin at the door and continue in the waiting area, corridor, car ride, and home that evening.

The point is not to turn every radiotherapy visit into a family conference. Not every patient wants family involved to the same degree, and not every caregiver can participate in the same way. Patient preference, privacy, and local workflow all matter. But individualized involvement is different from default exclusion. A supportive care approach in radiotherapy should at least assume that caregivers may need orientation, brief updates, and acknowledgment, and then tailor involvement to the patient and family rather than leaving it to chance. That becomes even more important when information needs, language, or cultural context may affect how confidently families can carry care forward after the patient leaves the department (Yuen et al. 2024).

This does not require a new program or additional staffing. More often, it requires better habits at a few predictable points in care. Before the first fraction, the team can give the caregiver a short practical orientation: what the treatment day usually looks like, why delays happen, and which symptoms or practical problems are worth reporting afterward. During a long course, if a session is substantially delayed, if the plan changes, or if treatment is interrupted, a brief update to the caregiver – when the patient agrees – can reduce avoidable uncertainty. When a course finishes, is replanned, or stops earlier than expected, a short closing explanation can help families understand what has changed, what comes next, and what kind of help the patient is likely to need at home. These are small interventions, but they directly address the uncertainty, information gaps, and unrecognized needs repeatedly described in the caregiver literature (Bratches et al. 2024; Oliveira et al. 2024; Yuen et al. 2024).

In everyday radiotherapy practice, waiting outside the treatment room is not empty time. It is one of the recurring places where family caregiving happens. Recognizing that fact does not stretch supportive care beyond reason. It corrects an omission long built into the way radiotherapy is organized and discussed, and it gives clinical teams a practical place to do better for both patients and families. In a field built around repeated attendance and repeated cooperation, that is not a sentimental add-on. It is part of taking the treatment course seriously as a lived experience.
